# Tensile effects in peridynamic plates with circular holes

**DOI:** 10.1007/s11012-026-02139-x

**Published:** 2026-06-12

**Authors:** Sofia Damian, Riccardo Cavuoto, Nicola M. Pugno, Massimiliano Fraldi, Luca Deseri

**Affiliations:** 1 Department of Civil, Environmental and Mechanical Engineering, University of Trento, via Mesiano 77, 38123 Trento, Italy; 2 Department of Structures for Engineering and Architecture, University of Naples “Federico II”, via Claudio 21, 80125 Naples, Italy; 3 Department of Neurosciences, Reproductive Sciences and Dentistry, University of Naples “Federico II”, via Claudio 21, 80125 Naples, Italy; 4 Mechano-X Labs, University of Trento, via Mesiano 77, 38123 Trento, Italy; 5 School of Engineering and Materials Science, Queen Mary University of London, Mile End Road, London, E1 4NS UK

**Keywords:** Nonlocality, Stress, Plates, Peridynamics

## Abstract

Microstructures are often responsible of the mechanical behavior at the macroscale level, governing damage processes and fractures. A suitable choice for modeling these kind of phenomena is a nonlocal theory, which considers microscale behaviors by defining an internal length scale. Thanks to this intrinsic property and to their ability to naturally handle discontinuities and singularities, nonlocal models are succeeding in analyzing onset of damage and fracture development in both previously unexplored fields and in well-known classical problems. Among these, this work aims to investigate the influence of a particular choice of nonlocal microstructure on a classical (local) problem of fracture propagation, namely that of a plate with a circular hole placed under tension. The selected microstructure corresponds to the discrete lattice representation of bond-based peridynamics, the theory on which this study is based. In particular, a recently proposed nonlocal dimensionally reduced model, crucial for minimizing the significant computational efforts associated with nonlocal settings, has been employed. The holed nonlocal plate has been analyzed for two different microstructures, each one corresponding to a proper choice of the horizon, a specific parameter representing the internal length scale of the medium. The analyses have been performed by keeping the same overall elastic stiffness, by computing the “peridynamic stress”, and by using a stress-based bonds’ failure criterion. The outcomes of this research highlight the shift of the stress peak, which becomes more distal when the internal length scale increases. In addition, the stress concentration area becomes more diffuse when the horizon rises, and the actual value of the stress concentration factor decreases. These results are in agreement with the development of the delamination surface, which is more diffuse when the horizon is higher.

## Introduction

Nonlocality has a key role in the mechanical behavior of the majority of natural materials, furthermore it plays a crucial function in design of architected materials, where some special features can be obtained thanks to a suitable use of nonlocal interactions. In many cases, these kind of long-range interactions lead the fracture process, thus determining unexpected onset of damage and fracture propagation (see e.g. [2, 8, 9, 20] ). For this reason, the study of benchmark fracture mechanics problems with changes in their microstructure may provide novel results and suggest innovative design strategies.

In the past, different methods have been used to study such kind of problems, but most of them rely on local theories and the study of fracture is made possible thanks to an enhancement of classical continuum mechanics (CCM) (see e.g. [4, 18, 29]). In the last century, several nonlocal theories have been developed to overcome the issues associated to these methods and to allow a more natural prediction of crack nucleation (see e.g. [12, 13, 33]). Among these, a recent strongly nonlocal theory called Peridynamics (PD) [38–40] demonstrates to be suitable for (i) dealing with fractures and (ii) for easily manage the internal length scale of the media. The possibility of naturally predict crack is guaranteed by the absence of specific requirements on the differentiability of the displacement field, which allows to handle the continuous and discontinuous part of the medium in the same way not asking for additional strategies in proximity of the onset of damage. For this reason, the equilibrium equations of the PD theory are written in an integro-differential formulation, and a key part is done by the setting of the degree of nonlocality. This is managed through a specific parameter called *horizon* δ, which corresponds to the internal length scale of the medium, and represents the maximum distance of two connected particles. Thanks to these properties, the PD theory has been widely used for several applications, e.g. (i) for modeling different kinds of material failures, as damages and fatigue (see e.g. [19, 25, 41]), (ii) for studying impact problems (see e.g. [39, 44]), and (iii) for dealing with multiscale problems (see e.g. [1, 6]). In some of these analyses, also classical fracture mechanics problems have been taken into account. As an example, some studies can be found for the case of a plate with a central hole (see e.g. [6, 15, 21, 28] ), but in most of these the PD approach has been used for predicting fracture in an almost local set up. Our aim is to take advantage of the PD theory to investigate the role of nonlocality in consolidated results, taking into account classical problems with different microstructures and nonlocality features.

By using the bond-based version of the PD nonlocal theory, this work aims to explore the influence of the degree of nonlocality in the onset of damage and “stress” distribution of a plate with a through-thickness central circular hole, subject to traction on its upper and lower planes. To do so, because PD involves internal forces only, a stress-like tensor field must be reconstructed according to the formulations presented in [15, 24]. Furthermore, as the integro-differentiable equilibrium equations of this theory may result in important computational efforts, the use of a dimensionally-reduced model is preferable. To this extent, the analyses have been conducted by using the model developed in [8, 9], which actually allows to capture crack nucleation in the thickness of the plate despite its dimensional reduction along the same direction. The final set up of the problem results to be that of plate with a central circular hole subject to tensile tractions, applied through imposed suitable displacements. Thanks to the symmetries, only a quarter of the whole plate has been analyzed. In this way, two novel and not real edges have been created, cutting a consistent number of bonds and leading to a localized loss of stiffness. In order to restore the initial conditions, the auxiliary nodes technique has been used, where real nodes of the plate outside the studied part have been taken into account to complete the horizon of the nodes along the novel edges. Due to symmetry, the displacements of the auxiliary nodes are determined by those of the real ones. In this fashion, no further unknowns are added to the problem itself and the undesired degradation of the stiffness is suitably managed. For what concerns the boundaries on the external perimeter of the plate, in the literature, many techniques have been proposed to deal with their nonlocal nature [23], which is responsible for a softening of the material near its edges, and does not allow to retrieve the classical local behavior. Some of these methodologies require to adapt the constitutive properties of the model near the boundaries, e.g. the volume method [5], the energy method [28, 30], the force normalization method [27], and the force density method. Other strategies, instead, focus on restoring the horizon near the edges, e.g. the variable horizon method [6, 10], and the fictitious node method [3, 26, 36]. In this work, we did not use any of these techniques for the mitigation of the loss of stiffness on the natural boundaries of the plate, as in this specific context we are actually interested in the behavior properly due to these nonlocal features, not in mimic the local continuum solution. For the same set up, two degrees of nonlocality have been considered, and a stress-based failure criterion for bonds’ breakage has been used. The analyses have been performed by considering the plates to have the same overall elastic stiffness. The outcomes provide novel insights about the location of the onset of damage and the stress distribution. On the one hand, we verified that by increasing the degree of nonlocality the onset of damage arises in a different place with respect to the usual location, so moving away from the tip of the hole as the horizon increases. On the other hand, the stress distribution inside the plate has been investigated. To this extent, we first made an in-depth analysis of the real meaning of stress in the context of a PD discretized continuum, thus starting from what found in [24] and adapting it to the necessities of a discretized approach. We derived a measure based on geometrical and static considerations, verifying its correspondence to that defined by Fallah et al. in [15], then we compared the results obtained for different degrees of nonlocality. The method has been tested by verifying the correspondence of the PD results for the smaller value of the horizon to those of the CCM. The results retrieved in this work are innovative under both the light of the location of the onset of damage and the stress distribution inside the plate, preceding possible usage for novel design strategies of architected materials, where the role of the degree of nonlocality has been shown to be non-negligible.

The structure of the paper is as follows. Section 2 describes the framework of the PD theory, focusing on the dimensionally-reduced model and on the boundary conditions applied. In Sect. 3 we provide the results retrieved through the analyses, starting from the distality of fracture, considering then stress distributions, finally introducing a comparison with the local continuum. Then, in Sect. 4 we illustrate the conclusions of this research.

## Model formulation

**Fig. 1:**
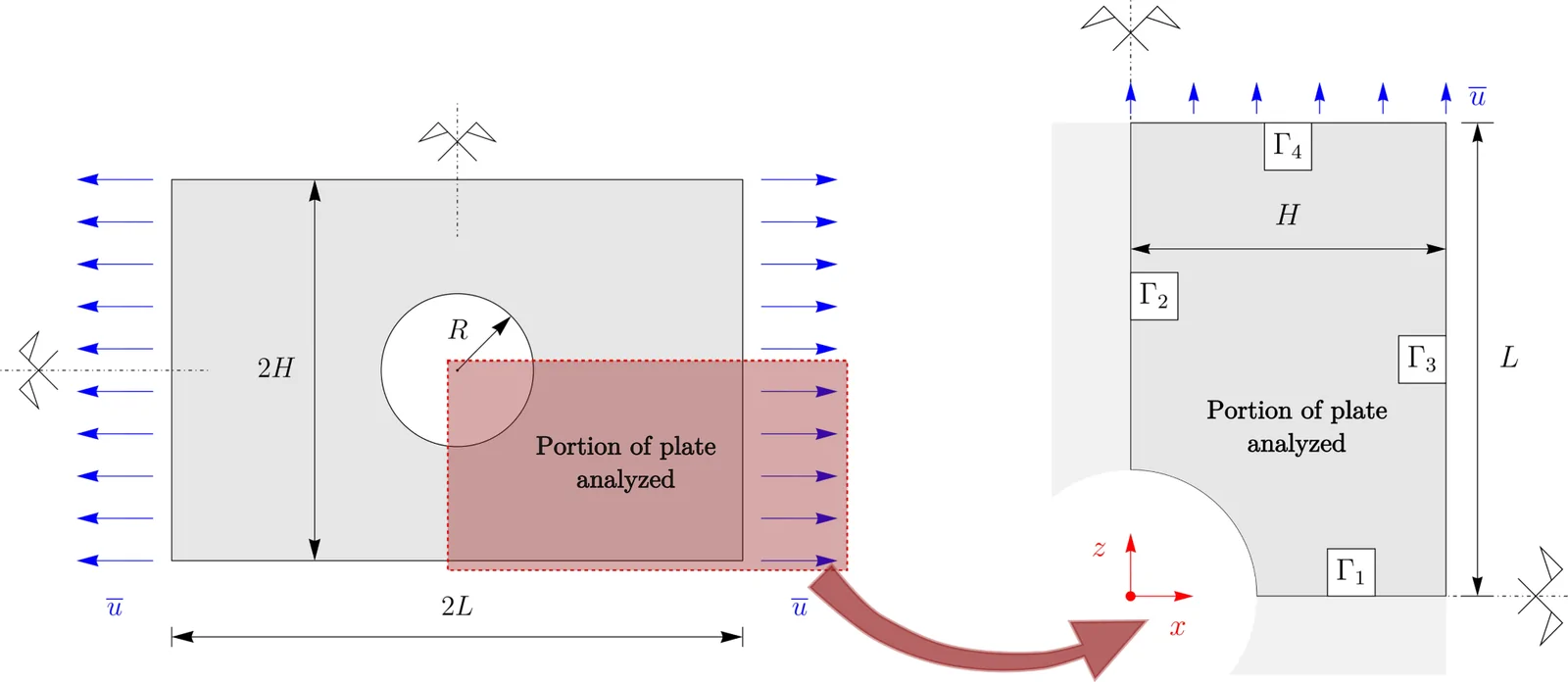
Plate with a circular hole subject to a traction test, which has been performed by applying a fixed increment of displacements. The red dashed line in the left-hand side picture shows the portion of the domain under exam. It is worth noting that, since the plate is nonlocal, the studied domain includes a larger portion of the plate than its quarter (figure on the right, grey shaded areas); indeed, auxiliary nodes on the left of $\Gamma _2$ and below $\Gamma _1$ are also involved in the analysis. The imposed displacement has been incrementally applied until the final value $\overline{u}=H/100$

The present study focuses on the investigation of the mechanical behavior of a nonlocal plate with a central circular hole subject to an imposed traction, for different degrees of nonlocality. The plate has been described with the model proposed in [8, 9] and it is represented by a rectangle of length 2*L*, height 2*H*, and unitary thickness. The hole characterizing the section has radius *R*. Equal and opposite displacements $\overline{u}$ have been imposed on the two opposite edges as shown in Fig. 1, on the left.

Due to the symmetry of the geometry and boundary conditions, it is possible to focus the study on one portion of the whole domain, considering a quarter of the plate, see Figs. 1 and 5. In this fashion, the analyses are performed on a rectangle of length L=1.5H, height *H*, unitary thickness, and a hole represented by a quarter of the circle of radius R=2H/5. Nevertheless, due to the nonlocality of the plate, the symmetry conditions around the boundary $\Gamma _1$ (see Fig. 1) are not simply reproducible, and some suitable strategies have to be considered, as further explained below.

**Fig. 2:**
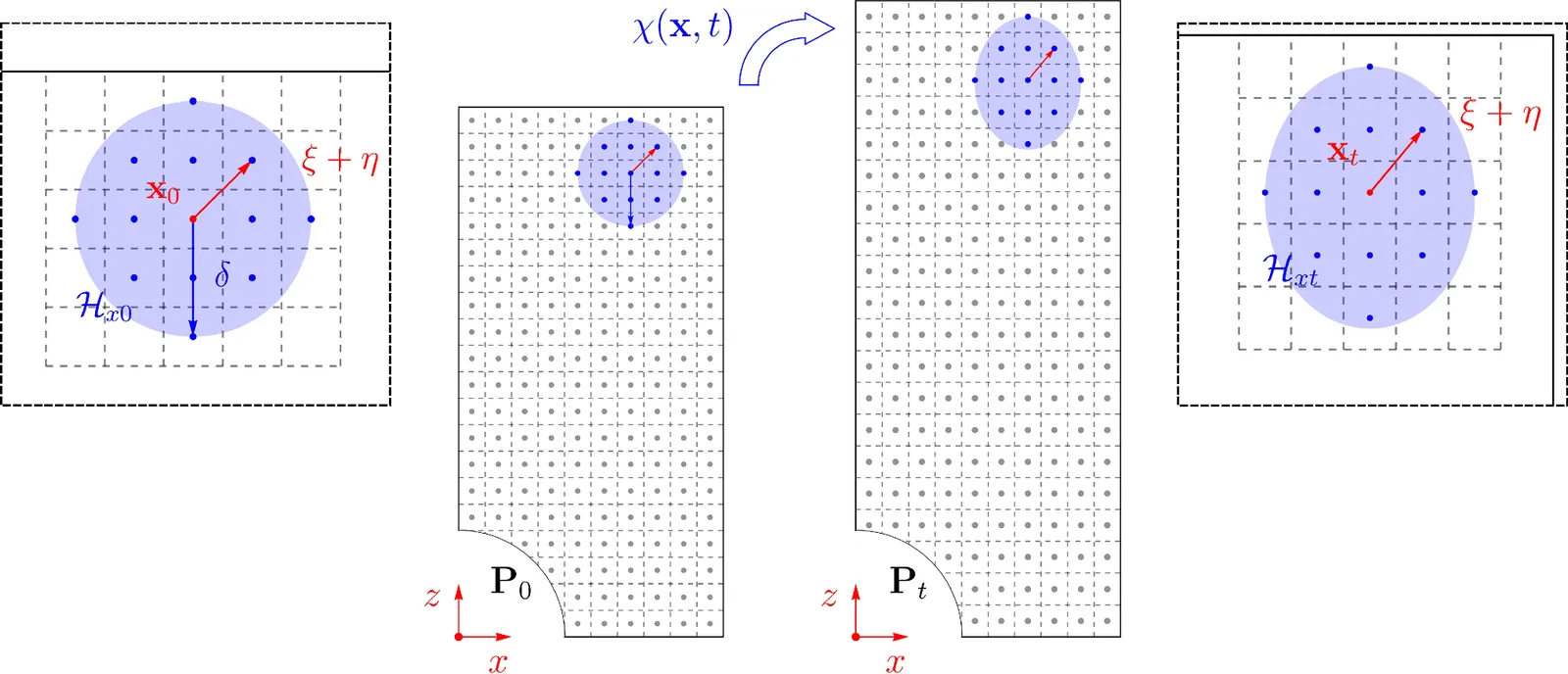
A quarter of the undeformed plate $\textbf{P}_{\textrm{0}}$ is subject to a certain loading condition which leads, at time *t*, to the deformed quarter of plate $\textbf{P}_{\textrm{t}}$. The family of points of $\textbf{x}_{\textrm{0}}$, $\mathcal {H}_{\textrm{x0}}$ defined as a circle of radius δ, is also mapped into the novel configuration as $\mathcal {H}_{\textrm{xt}}$. The points enclosed in the horizon of $\textbf{x}_{\textrm{0}}$ are all and only the ones interacting with $\textbf{x}_{\textrm{0}}$ through pairwise forces

**The Model** The plate has been modeled with the recently developed bond-based PD [38] and, in particular, with the dimensionally-reduced PD model proposed in [8, 9]. According to this choice, the plate has been described by a grid of uniformly spaced material particles each of which represents a portion of the plate’s mass or volume, see Fig. 2. In agreement with the general theory, these particles are connected one another through massless spring-like bonds if and only if their relative distance is enclosed in a circle of radius δ, called horizon, see Fig. 2. The latter parameter allows one to set the desired nonlocality so to model the long-range interactions between material points. The equilibrium equations (static case) are written in the integro-differential form as follows:

1 $$\begin{aligned} \int _{\mathcal {H}_x} {\textbf{f}(\boldsymbol{\xi },\boldsymbol{\eta }) \, dV_{\mathrm {\mathbf {x'}}}}+\textbf{b}(\textbf{x})=0 \; . \end{aligned}$$

Here, the integration of the *pairwise force*, $\textbf{f}(\boldsymbol{\xi },\boldsymbol{\eta })$, is performed within the domain $\mathcal {H}_x$, the family of points connected to $\textbf{x}$, while $\textbf{b}(\textbf{x})$ is the vector of the external body forces. The pairwise force is carried by the bond connecting two particles $\textbf{x}$ and $\mathbf {x'}$, whose relative distance and relative displacement are $\boldsymbol{\xi }$ and $\boldsymbol{\eta }$, respectively. For a standard microelastic PD material the definition of that force is given by the derivative of the *pairwise potential function* $w(\boldsymbol{\xi },\boldsymbol{\eta })$, namely:

2 $$\begin{aligned} \textbf{f}(\boldsymbol{\xi },\boldsymbol{\eta })=\frac{\partial w (\boldsymbol{\xi },\boldsymbol{\eta })}{\partial \boldsymbol{\eta }}=\mu (s) \; c \; s(\boldsymbol{\xi },\boldsymbol{\eta })\; \frac{|\boldsymbol{\xi }|^2}{\sigma (\boldsymbol{\xi })} \boldsymbol{\xi } \; , \end{aligned}$$

where *c* is the *bond constant*, which represents the bonds’ stiffness, *s* is their stretch defined as $s(\boldsymbol{\xi },\boldsymbol{\eta })=|\boldsymbol{\eta }|/|\boldsymbol{\xi }|$, and $\sigma (\boldsymbol{\xi })=|\boldsymbol{\xi }|^3$ is a function ensuring the integrability of Eq. (2). Finally, the parameter μ(s) allows for naturally considering damage and fracture occurrences by giving value 1 when the considered bond’s stretch is lower than or equal to the threshold $s_{\textrm{cr}}$, value 0 otherwise. In this specific case of study, rather than referring to a critical elongation criterion, a stress-based one is employed and thoroughly discussed in what follows. As mentioned before, a stress-like field is reconstructed from the performed PD analyses by using the approaches introduced in [15, 24].

The model developed in [8] is based on Eqs. (1) and (2), and it is characterized by some assumptions, necessary to deal with the dimensional reduction. First, the following ansatz for the main unknown, namely the displacement field, is chosen:

3 $$\begin{aligned} \textbf{u}(x,z)=\textbf{u}_{\textrm{a}}(x,z)+\textbf{u}_{\textrm{J}}(x,z) \; , \end{aligned}$$

where $\textbf{u}_{\textrm{a}}(x,z)$ is its absolutely continuous part, and $\textbf{u}_{\textrm{J}}(x,z)$ is the corresponding “ jump” part, which allows for recognizing through-thickness damage and delamination. The first term is described by an appropriate polynomial expansion around the midplane of the plate; it is worth noting that the polynomial’s degree is chosen on the basis of the desired accuracy, of the degree of nonlocality, and of the kind of loading condition imposed:

4 $$\begin{aligned} \textbf{u}_{\textrm{a}}(x,z) \simeq \pmb {\mathscr {A}} (x) + \pmb {\mathscr {B}} (x) z + \pmb {\mathscr {C}} (x) z^2 + ... \; . \end{aligned}$$

On the other side, $\textbf{u}_{\textrm{J}} (x,z)$ is given by the product of the jump $\textbf{j}(x)$ and the Heaviside function whose argument depends on the delamination surface *h*(*x*), namely:

5 $$\begin{aligned} \textbf{u}_{\textrm{J}}(x,z)=\textbf{j}(x) \cdot \Theta \left( z-h(x)\right) \; . \end{aligned}$$

It is worth noting that the jump vector $\textbf{j}(x)$ is a function of the sole variable *x*, due to the plane strain configuration of the problem. It is due to the delamination process and it represents the inelastic part of the displacement field, corresponding to the distance between the fracture surfaces.

In this work, we use the model to study the mechanical response of a nonlocal plate with a circular hole. Both the absolutely continuous part of the displacement field and the jump part are taken into account, in order to detect damage and delamination occurrences. The geometrical configuration of this problem with respect to the original one, where the model was first applied [8], is particularly different, thus a clarification of the use of the reduction procedure is provided in what follows. This is a plane strain problem, the plate is supposed to have a homogeneous behavior in the *y*-direction, thus only a unitary strip along *y* is considered, while the investigation focuses on the (*x*, *z*) plane. Differently from [8], the reduction plane is not located at half the thickness of the thin plate, but rather on the *x*-axis, thus the reduction is performed in *z*, where the plate cannot be considered thin. Despite this, the model, supported by a proper choice of the ansatz, is able to reproduce the variability of the mechanical behavior not only along *x*, but also in the *z*-direction, as it will be shown in the results of this work. In this fashion, a FEM analysis of the local continuum allowed us to define the more suitable absolutely continuous part of the displacement field . For this purpose, the FEM model coincides with the discrete bond-based PD lattice, where bonds are represented by springs and each node is connected with all and only the nodes enclosed in the distance of its horizon. The mechanical characteristics have been derived with respect to the PD constants, and the sole elastic regime has been investigated. The loading mode and boundary conditions were the same as in the PD problem, and the analyses have been conducted for different values of the horizon. The values of both $u_x$ and $u_z$ have been represented as functions of *z* for each of these cases, and this allowed us to verify that no substantial differences can be seen in the polynomials degree of the displacement components for different degrees of nonlocality. In this fashion, through a polynomial interpolation of the functions $u_x$ and $u_z$, a fourth order polynomial ansatz has been chosen for the x-component, and a third order one for the *z*-component. The expansion is performed along the *z*-axis, and the coefficients are linearly dependent on *x*, i.e.

6 $$\begin{aligned} \begin{aligned} \textbf{u}(x,z)=\{&a(x) + b(x) z + c(x) z^2 + d(x) z^3 + e(x) z^4, \\  &f(x) + g(x) z + m(x) z^2 + n(x) z^3 + j(x) \cdot \Theta (z - h(x)) \} \; . \end{aligned} \end{aligned}$$

The location of the delamination surface *h*(*x*) has been set a priori, as explained below. The jumps *j*(*x*) are assumed to be non-negative in order to satisfy the condition of non-interpenetration of the matter. It can be noted that, with respect to the general definition of the jump part of the displacement field, Eq. (5), here the sole *z*-component of $\textbf{j}(x)$ has been taken into account. This is due to the fact that this type of problem leads to mode I of fracture, thus no jumps in the matter are allowed in the *x*-direction.

The solution of the system is obtained through the minimization of the *pairwise potential energy*, which has been written in the reduced formulation proposed in [8]:

7 $$\begin{aligned} \omega _{red}=\int _{0}^{L} \int _{0}^{L} {\omega (\boldsymbol{\xi },\boldsymbol{\eta }) \, dz \, dz'} \; , \end{aligned}$$

where the symbol ’ represents a point belonging to the family of $\textbf{x}$, and the pairwise potential energy is the one defined in [38]:

8 $$\begin{aligned} \omega (\boldsymbol{\xi },\boldsymbol{\eta })=\int {\textbf{f}(\boldsymbol{\xi },\boldsymbol{\eta }) \cdot d\boldsymbol{\eta }} \; . \end{aligned}$$

**Stress interpretation.** It is well known that one of the most important differences among CCM and the PD theory is that this latter has no particular requirements on the differentiability of the displacement field. This allows one to use the same mathematical formulation both for the continuous and the discontinuous part of the solids, but it requires the equilibrium to be written as an integro-differential equation. Due to this mathematical framework, the main outcome of a PD simulation is the displacement field, not stresses and strains as in CCM. However, starting from the displacement field itself, stresses can be reconstructed using the techniques proposed in [15, 24]. Indeed, the distribution of the stresses inside the plate under consideration is derived through the discretization of the approach in [24], as proposed in [15]. The PD stress tensor $\boldsymbol{\sigma }(\textbf{x})$ of a generic point $\textbf{x}$, first introduced in [24], can be written as follows:

9 $$\begin{aligned} \boldsymbol{\sigma }(\textbf{x})=\frac{1}{2} \int _{\Sigma } \int _0^\infty \int _0^\infty (y+z)^2 \; \textbf{f}(\textbf{x}+y\hat{\textbf{m}},\textbf{x}-z\hat{\textbf{m}}) \otimes \hat{\textbf{m}} \, dz \, dy \, d\Omega _m \; . \end{aligned}$$

Here, Σ is the unit sphere centered in $\textbf{x}$. The pairwise force function $\textbf{f}$ is first computed for the generic points $\textbf{x}'=\textbf{x}+y\hat{\textbf{m}}$ and $\textbf{x}''=\textbf{x}-z\hat{\textbf{m}}$, then integrated in the unit sphere Σ through the variation of the solid angle $d\Omega _m$. The computation of each bond passing through $\textbf{x}$ is ensured by the integration of the variables *y* and *z*, which are scalars identifying the position of $\textbf{x}'$ and $\textbf{x}''$ with respect to $\textbf{x}$, along the direction of the unit vector $\hat{\textbf{m}}$. As proved in [24], the divergence of (9) is null, so this kind of measure corresponds to the Cauchy stress tensor. As described in [15, 24], the traction vector $\boldsymbol{\tau }(\textbf{x},\hat{\textbf{n}})$ is given by the scalar product among the PD stress tensor (9) and the normal $\hat{\textbf{n}}$ to the plane π crossing the bond considered:

10 $$\begin{aligned} \boldsymbol{\tau }(\textbf{x},\hat{\textbf{n}})=\frac{1}{2} \int _{\Sigma } \int _0^{\infty } \int _0^{\infty } (y+z)^2 \; \textbf{f}(\textbf{x}+y\hat{\textbf{m}},\textbf{x}-z\hat{\textbf{m}}) \; \hat{\textbf{m}} \cdot \hat{\textbf{n}} \, dz \, dy \, d\Omega _m \; . \end{aligned}$$

This can be proved by computing the force exerted by the infinitesimal volume of $\textbf{x}'$, $dA_{x'} \, dy$, on the infinitesimal volume associated to $\textbf{x}''$, $dA_{x''} \, dz$, as follows. First, the two infinitesimal areas, $dA_{x'}$ and $dA_{x''}$, can be defined as the differential areas of a sphere of radius (y+z) subtending the solid angle dΩ, and centered in $\textbf{x}''$ and $\textbf{x}'$, respectively (see Fig. 1 in [24]):

$$\begin{aligned}&dA_{x'}=(y+z)^2 \; d\Omega  &   dA_{x''}=(y+z)^2 \; d\Omega \; . \end{aligned}$$

Then, the infinitesimal area $dA_{\pi }$ is determined by the intersection among the plane π of normal $\hat{\textbf{n}}$, and the cylinder with cross-sectional area $dA_{x'}=dA_{x''}$ and direction $\hat{\textbf{m}}$ connecting $\textbf{x}'$ and $\textbf{x}''$. Due to geometrical considerations, the intersection area can be written as follows:

$$\begin{aligned} dA_{\pi }=\frac{dA_{x'}}{\hat{\textbf{m}}\cdot \hat{\textbf{n}}}=\frac{(y+z)^2 \, d\Omega }{\hat{\textbf{m}}\cdot \hat{\textbf{n}}} \; , \end{aligned}$$

thus the differential force per unit area on the plane π reads:

11 $$\begin{aligned} \frac{d\textbf{t}(\textbf{x},\hat{\textbf{n}})}{dA_{\pi }}=\frac{\textbf{f}(\textbf{x}',\textbf{x}'') \, (y+z)^4 \, d\Omega ^2 \, dy \, dz}{(y+z)^2 \, d\Omega } \, \hat{\textbf{m}} \cdot \hat{\textbf{n}} \; . \end{aligned}$$

**Fig. 3:**
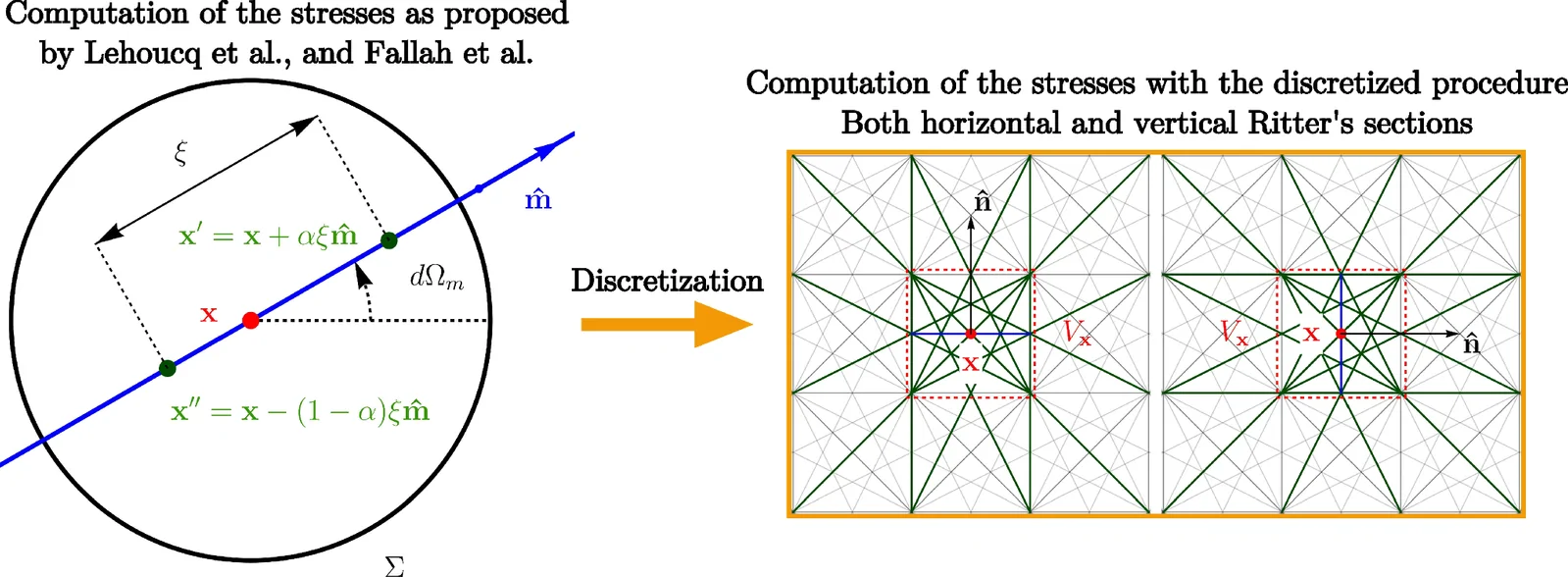
Representation of the equivalence of the discretized method for the derivation of the stresses with the one proposed in [15, 24]. On the left-hand side of the image, the schematic of the continuum approach proposed in [24] for the case of a 2D-continuum is reported. It is worth noting that, in this case, the sphere is reduced to a disk, and the solid angle to a plane angle, moreover, the point of application is $\textbf{x}$ and the segment’s nodes are described with the variables ξ=y+z and $\alpha =y/\xi$. On the right-hand side of the picture, the discretized method applied in this study is represented. Both the cases of horizontal and vertical Ritter’s sections for the point $\textbf{x}$ have been proposed here

This proves the relation (10), where half of the value is assumed because the same bond is computed twice. The result reported in (11), obtained by the Authors for the PD continuum, has been adapted in [15] to the discretized medium, and re-proposed here using a geometrical interpretation, see the representation provided in Fig. 3. To this extent, the concept of Ritter’s sections has been employed for identifying the bonds contributing to the stress tensor of a generic point $\textbf{x}$.

Indeed, Eqs. (18)–(24) in [15] explain the transition from the continuum PD stress tensor to the discrete one. In the following, a revised formulation adapted to the notation of this work is proposed. First, Eq. (9) is re-written substituting the variables *y* and *z*. The vector $\boldsymbol{\xi }$ represents the relative distance of the points connected by a bond, as in the usual peridynamic formulation, and a novel parameter α is introduced to indicate the position of the bond with respect to $\textbf{x}$, i.e., ξ=y+z and $\alpha =y/\xi$

12 $$\begin{aligned} \boldsymbol{\sigma }(\textbf{x})=\frac{1}{2} \int _{\Sigma }{\int _0^\infty {\int _0^1 {\xi ^2 \; \textbf{f} \left( \textbf{x}+\alpha \xi \hat{\textbf{m}}, \textbf{x}-(1-\alpha )\xi \hat{\textbf{m}} \right) \otimes \left( \xi \hat{\textbf{m}}\right) \; d\alpha } \; d\xi } \; d\Omega _m} \; . \end{aligned}$$

It is worth noting that, in this 2D problem, Σ represents the unit disk for the integration of the angle $\Omega _m$. Then, the continuum is discretized through a tessellation, thus the area of the plate is divided into identical squares, called Voronoi cells. This is done using the grid with horizontal step $\Delta _x$ and vertical step $\Delta _z$. Every generic property of each cell is due to the bonds crossing its surface, the average of their contribution is entitled to represent the generic property of this portion of area, which is supposed to be concentrated in the point in the center of the square. From a mathematical point of view, a point $\textbf{x}$ represents its associated Voronoi cell $C_{\textbf{x}}$, whose area $S_{\textbf{x}}=\Delta _x \cdot \Delta _z$ can also be seen as a function of

13 $$\begin{aligned} \Delta _{\textbf{x}} (\hat{\textbf{x}})= {\left\{ \begin{array}{ll} 1 \quad \quad \hat{\textbf{x}} \in C_{\textbf{x}} \\ 0 \quad \quad \hat{\textbf{x}} \notin C_{\textbf{x}} \end{array}\right. } \; , \end{aligned}$$

where $\hat{\textbf{x}}$ is a generic point in the plate, as $S_{\textbf{x}}=\int _{\mathbb {R}^2}{\Delta _{\textbf{x}} \; dS}$. The average of a generic property $g(\hat{\textbf{x}})$ is defined as follows:

14 $$\begin{aligned} \text {avg}\left[ g(\hat{\textbf{x}}) \right] = g(\textbf{x}) = \frac{1}{S_\textbf{x}} \int _{\mathbb {R}^2}{\Delta _{\textbf{x}}(\hat{\textbf{x}}) g(\hat{\textbf{x}}) dS} \; , \end{aligned}$$

and this allows for the discretization of the pairwise force functions, which is the required step for discretizing the PD stress tensor in Eq. (12). Indeed, by using Eq. (14) the discretization of the pairwise force among two generic particles $\hat{\textbf{x}_1}$ and $\hat{\textbf{x}_2}$, which correspond to two middle section points in our case, reads:

15 $$\begin{aligned} \textbf{f}(\hat{\textbf{x}_1},\hat{\textbf{x}_2})=\sum \limits _{\textbf{x}_1 \ne \textbf{x}_2} \frac{\textbf{F}(\textbf{x}_1 \, , \textbf{x}_2)}{S_{\textbf{x}_1} \, S_{\textbf{x}_2}} \Delta _{\textbf{x}_1}(\hat{\textbf{x}_1}) \, \Delta _{\textbf{x}_2}(\hat{\textbf{x}_2}) \; , \end{aligned}$$

where it is worth noting that the particles $\textbf{x}_1$ and $\textbf{x}_2$ mutually exchange the force $\textbf{F}(\textbf{x}_1,\textbf{x}_2)$, and that the sum over the indices of $\textbf{x}$ gives the total amount of force per unit volume carried by all the bonds connecting points in the volumes of $\mathbf {x_1}$ and $\mathbf {x_2}$. By replacing $\hat{\textbf{x}_1}$ with $\textbf{x}+\alpha \boldsymbol{\xi }$, and $\hat{\textbf{x}_2}$ with $\textbf{x}-(1-\alpha ) \boldsymbol{\xi }$, so assuming the point $\textbf{x}$ as the middle point of a section and considering the forces carried by all the bonds connecting points of its volume with any point of all the other surrounding volumes, one gets:

16 $$\begin{aligned} \textbf{f}(\textbf{x}+\alpha \boldsymbol{\xi } \, , \textbf{x}-(1-\alpha )\boldsymbol{\xi })=\sum \limits _{\textbf{x}_1 \ne \textbf{x}_2} \frac{\textbf{F}(\textbf{x}_1 \, , \textbf{x}_2)}{S_{\textbf{x}_1} \, S_{\textbf{x}_2}} \Delta _{\textbf{x}_1 -\textbf{x}_2}(\boldsymbol{\xi }) \, \Delta _{\textbf{x}_1+\alpha (\textbf{x}_2-\textbf{x}_1)}(\textbf{x}) \; . \end{aligned}$$

In this fashion, the PD stress tensor obtained in [15] for each particle $\textbf{x}$, i.e.

17 $$\begin{aligned} \boldsymbol{\sigma }(\textbf{x})=\frac{1}{2 S_{\textbf{x}}} \sum \limits _{\hat{\textbf{x}} \ne \textbf{x}} \textbf{F}(\hat{\textbf{x}} \, , \textbf{x}) \otimes (\hat{\textbf{x}}-\textbf{x}) \; . \end{aligned}$$

**Fig. 4:**
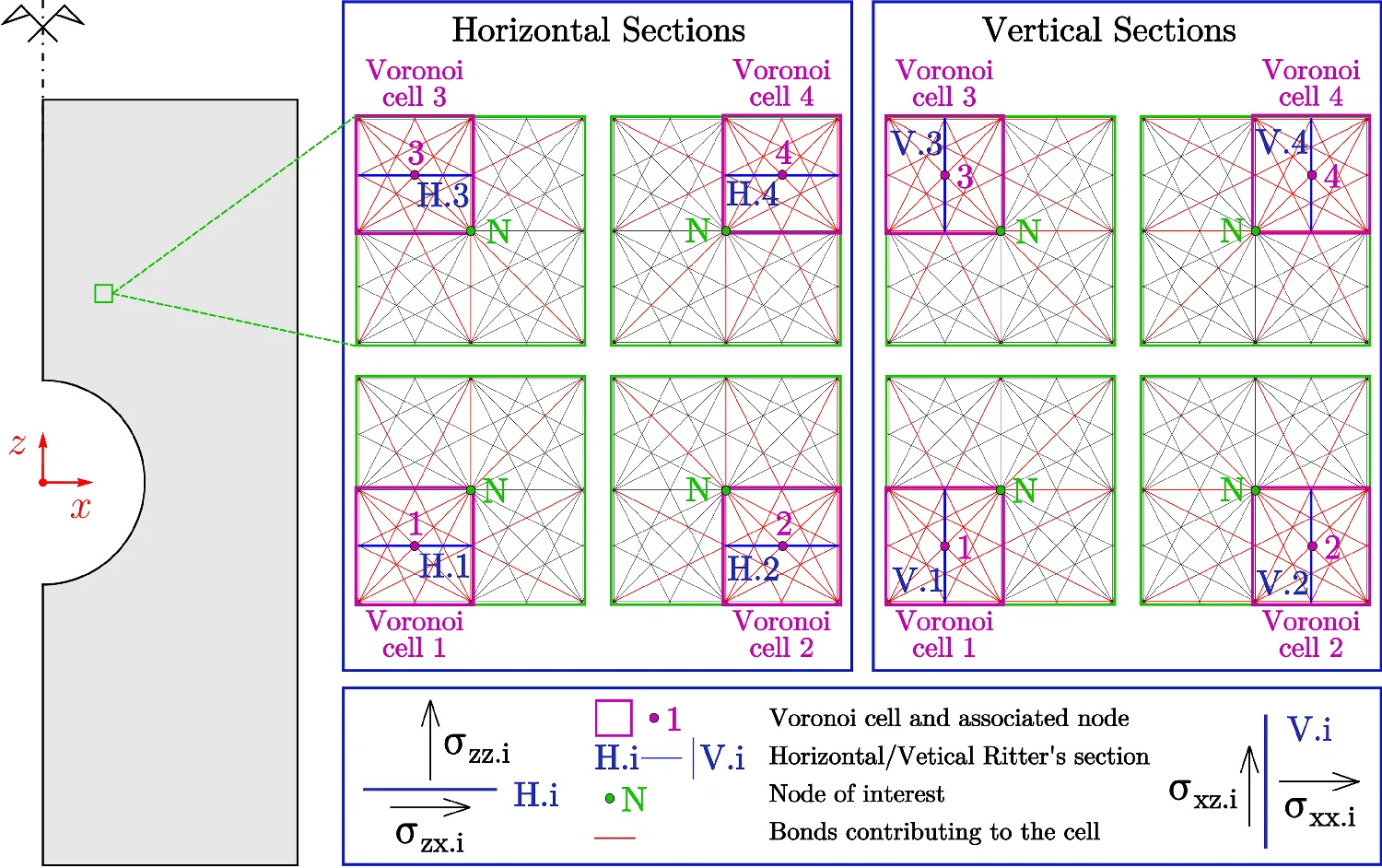
Example of horizontal and vertical Ritter’s sections used for computing the stresses in the node N. In the picture on the right, the green boxes correspond to the magnification of the 4 Voronoi cells surrounding a generic node N of the plate. The box is reported 4 times to represent the horizontal sections associated to the 4 cells, i.e. H.1, H.2, H.3 and H.4, and other 4 times for the vertical sections, i.e. V.1, V.2, V.3 and V.4. As described in the legend, for each Voronoi cell the horizontal sections allow for computing the stresses $\sigma _{zz}$ and $\sigma _{zx}$, while $\sigma _{xx}$ and $\sigma _{xz}$ are retrieved starting from the vertical sections. The stress tensor components in N are obtained by averaging the corresponding stresses derived in the surrounding Voronoi cells

From a practical point of view, these results, specifically the PD stress tensor in Eq. (17), have been retrieved by using the geometric approach cited before. Thus, the identification of the bonds contributing to the mechanical properties of a generic Voronoi cell centered in $\textbf{x}$ is performed through the Ritter’s sections. These are defined as a pair of sections, an horizontal and a vertical one, both centered in $\textbf{x}$. The horizontal section has a total length of $\Delta _x$, covering the width of the cell, while the length of the vertical one is $\Delta _z$, including the entire height of the cell. In this fashion, the sections cross the cells intersecting all and only the bonds whose averaged properties characterize the point $\textbf{x}$. It can be noted that, by using this procedure, the factor 1/2 in Eq. (17) is not needed, as the bonds are already computed once. This method undoubtedly represents an approximation approach, for this reason both the horizontal and vertical sections are needed, as they give different and complimentary information. Indeed, each pair of Ritter’s sections leads to two distinct sets of bonds crossing the Voronoi cell under exam, and all of these contribute with their pairwise force to the determination of the stress tensor. In particular, the bonds cut by the horizontal section provides the *z* components, while those intersected by the vertical one supplies the *x* components, as specified in Fig. 4. In this fashion, the stress tensor has been determined in the central nodes of each Voronoi cell, while we are interested in the values associated to the vertices of these cells, which correspond to the nodes initially used for the discretization of the plate, e.g., the node *N* in Fig. 4. A generic node of the plate is shared as a vertex of four cells, on the edges it is common to two cells, while in the corners it is uniquely associated to a cell. An averaging procedure has been adopted for determining the stresses in the nodes of interest, thus the stress tensor components in each of these nodes are computed as the average values of the corresponding stress tensor components retrieved in the cells for which the node itself represents a vertex, i.e. the surrounding cells.

The exact procedure used for the computation is reported in Algorithm 1, and represented in Fig. 4.

**Algorithm 1:**
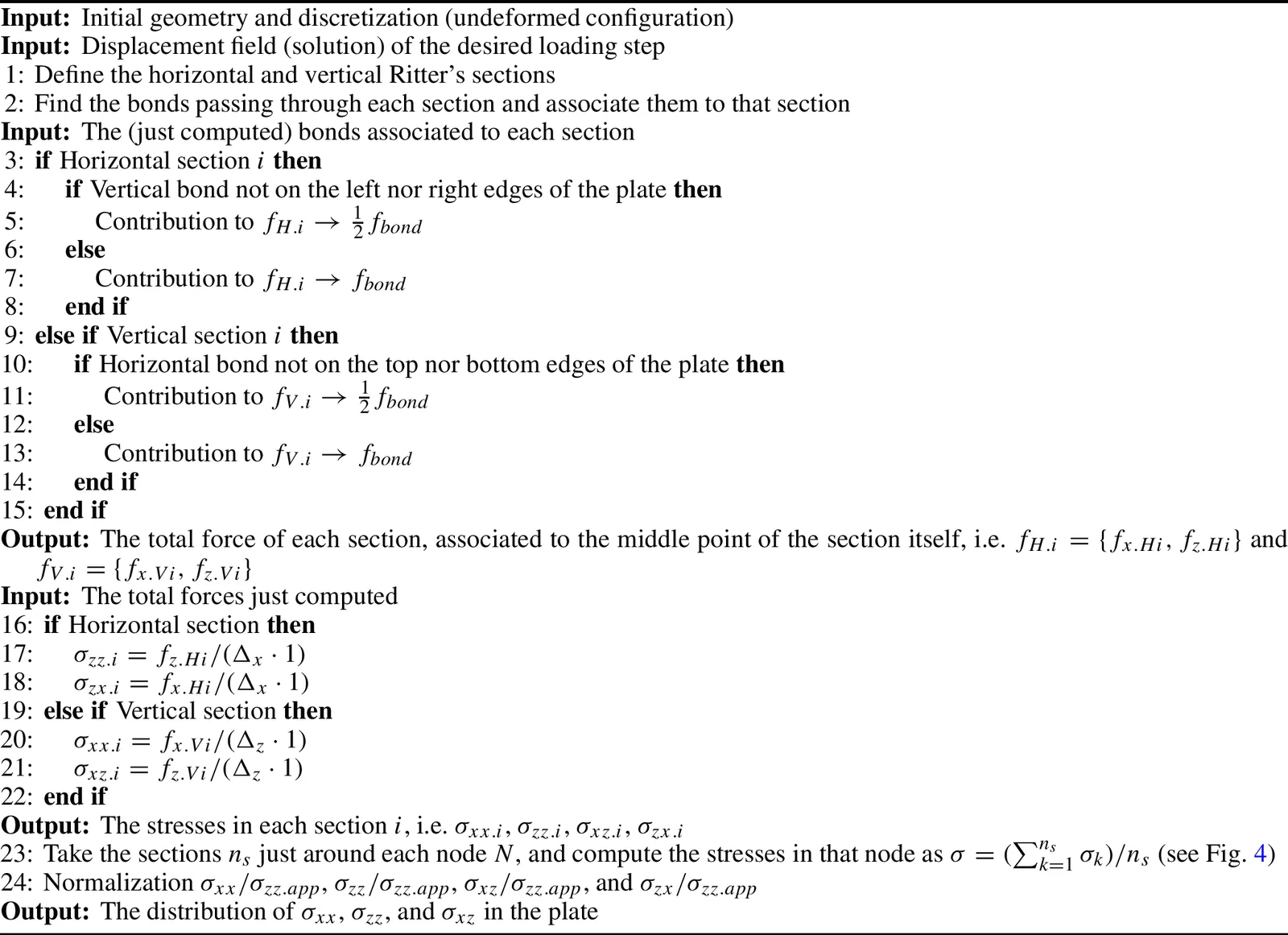
Description of the steps used for the computation of the stresses

It is worth noting that, in the steps described in lines 5 and 11 of Algorithm 1, only 1/2 of the bond’s contribution to the total pairwise horizontal and vertical forces is taken into account. The bonds we are referring to in these steps are, specifically, the shared vertical bonds on the left and right side of two adjacent cells, and the shared horizontal bonds on the top and bottom side of two adjacent cells. These are not bonds connecting the two cells, for which the full contribution has to be computed, but rather bonds that are common to two cells due to the discretization and to the specific choice of the Ritter’s sections. If we imagine to integrate the contribution obtained from all the horizontal Ritter’s section at a given coordinate *z* (or vertical at a given coordinate *x*), we should retrieve the total pairwise force in the *z* direction (in the *x* direction). For this reason, to be computed once, the contributions of these kind of bonds must be distributed between the cells sharing them. Here, the choice of the Authors is to equally divide them through the factor 1/2.

In the following paragraph the results obtained using the above described procedure are reported. The method has been tested by computing the stresses for an almost local case and by verifying that the stress concentration factor corresponds to the one derived through analytical computations and a FEM analysis. Then, the procedure has been used to investigate the distribution of the stresses in the plate under exam, for different degrees of nonlocality.

As correctly pointed out in [37], when the discretized PD model is used to approximate a local continuum, the stress formulation reported in this paragraph gives less accurate results on the boundaries, unless fictitious layers are considered. However, due to the specific purposes of this work, such correction methods have not been introduced. Indeed, we are interested in design strategies of plates that are characterized by a (specific) nonlocal microstructure, thus taking into account all the possible consequences of nonlocality on the mechanical responses of the plates themselves.

**Stress-based failure criterion.** The stretch-based bonds’ failure criterion was introduced in [39], and widely used in many peridynamic applications. In the following years other kind of failure criteria have been developed, based both on energy (see e.g. [31]) and stress considerations (see e.g. [11]). To correctly address experimental evidence on fracture nucleation and propagation in materials with strongly nonlocal microstructure (i.e., paper and, in general, materials made up of clusters of fibers), in which “distal” stress concentration and damage occur quite commonly, it was necessary to develop a criterion capable of associating the breakage of bonds to the areas where the stress peak is concentrated. Classical elongation and energy based criterion have proven to be strongly affected rather than by the stress, by the displacement field and failed at following the natural stress concentrations of the nonlocal microstructures. Due to these considerations, in the present work a stress-based bonds’ failure criterion is preferred. The stress $\sigma _{zz}$ has been computed for each node along the $\Gamma _1$ boundary with the method widely explained in the previous paragraph. When the value of $\sigma _{zz}$ in one node reached the critical value $\sigma _{cr}$, the “ jump” part of the displacement field was activated and the corresponding *j*(*x*) was added to the unknowns of the problem to be computed during the minimization of the reduced energy. The critical stress $\sigma _{cr}$ has been set to allow for a sufficient number of elastic loading steps before the bonds’ rupture. Indeed, in addition to the activation of the “ jump”, when $\sigma _{zz}$ went beyond $\sigma _{cr}$ all the bonds connected or passing through the corresponding node broke. This method, also reported in Algorithm 2, allowed us to deal with both fracture and distribution of stress through the same measure, while the stretch-based criterion provides a fracture growth area different from that of the stress peak.

**Algorithm 2:**
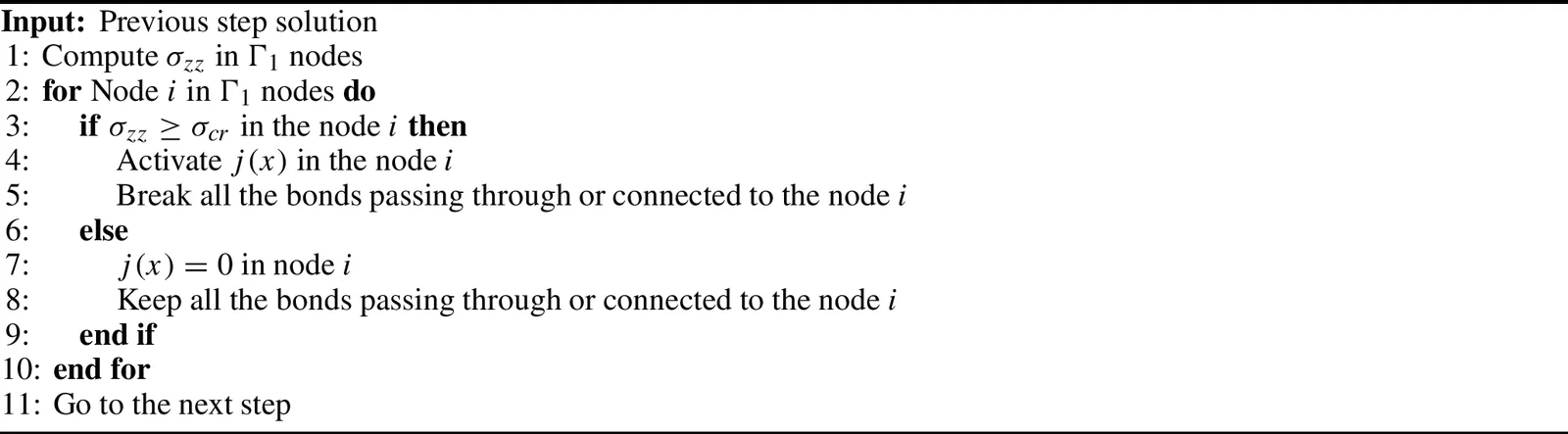
Stress-based failure criterion

In this specific context, the value of $\sigma _{cr}$ has been set for convenience in such a way that, for both the analyzed cases, a sufficient number of elastic steps is allowed before the onset of damage appears. Despite this, it is worth considering that $\sigma _{cr}$ is a parameter with a specific physical meaning, and it can be related to the bonds’ released energy, as well as to the global energy of the medium. In this fashion, the possibility of experimentally tuning the mechanical parameter of the model to reproduce a specific material is ensured by adapting $\sigma _{cr}$ to reproduce the value of its global energy.

**Fig. 5:**
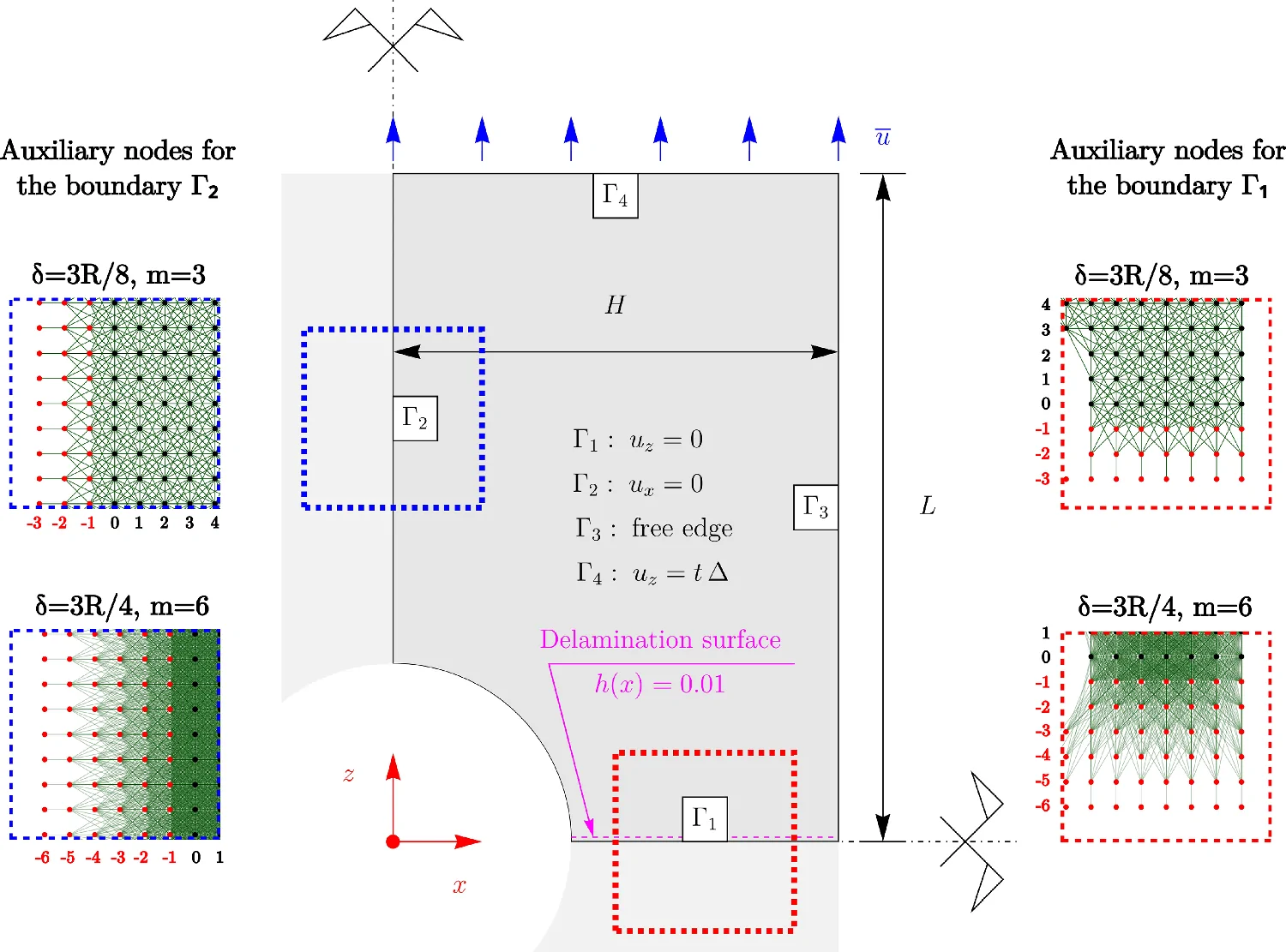
Portion of the plate represented in Fig. 1, subject of the analysis. The symmetry conditions are imposed on the $\Gamma _1$ and $\Gamma _2$ boundaries. The upper edge $\Gamma _4$ is subject to the application of the imposed displacement Δ for each loading step *t* until reaching the final displacement $|\overline{u}|$, while $\Gamma _3$ is a traction-free edge. The central figure represents the location of the boundaries $\Gamma _1$ and $\Gamma _2$ (details in the red and blue dashed squares, respectively), where the auxiliary nodes have to be added to restore the proper stiffness, as well as the location of the delamination surface. On left-hand and right-hand side of the image the enlarged details of the auxiliary nodes (red points) of $\Gamma _2$ and $\Gamma _1$ are represented

**Boundary Conditions.** The edges $\Gamma _1$ and $\Gamma _2$ are not real edges, delimiting the actual geometry of the plate like $\Gamma _3$ and $\Gamma _4$ but, rather, fictitious boundaries needed for the study of the quarter of the plate. The model is built on the bond-based PD theory. Hence, many of the bonds connecting the points on and around the edges $\Gamma _1$ and $\Gamma _2$ and the portion of the plate neglected in the study have been removed. This means that this fictitious boundaries has lost part of their stiffness, which must be restored. To overcome the softening of the boundaries of a PD continuum many methods have been proposed, see e.g. [23] and references cited therein. Here the fictitious nodes approach, first introduced in [16], is adopted, see Fig. 5. As initially stated, the geometrical symmetry along the boundary $\Gamma _2$ is the sole that can be simply exploited to reduce the computational efforts of the analyses. Indeed, the conditions prior to loading are recovered by imposing that the auxiliary nodes (i.e. real nodes of the plate outside the interested half studied) related to $\Gamma _2$ have the following imposed displacement:

18 $$\begin{aligned} \{u_{x,-i},u_{z,-i}\}=\{-u_{x,i},u_{z,i}\} \quad \quad \forall i=1,...,n_{aux.n.\Gamma _2} \, . \end{aligned}$$

Here the nodes along the $\Gamma _2$ edge are defined with the subscript “0”, for which $u_x=0$ is prescribed. On the opposite side of the domain under consideration, the definition of the symmetric condition on $\Gamma _1$ requires a different approach. The onset of damage is usually located in proximity of the stress concentration peak, and for this kind of problem this is expected to be nearby the tip of the hole [32], while the fracture should propagate perpendicularly to the direction of the applied loading condition, symmetrically with respect to the *x*-axis. For these reasons, $\Gamma _1$ is expected to be the site of the delamination surface. Despite this, the symmetric behavior is partially lost for two reasons. First, the Heaviside function that mathematically describes the delamination surface is not defined on z=0 where grid’s nodes are placed, thus the site of fracture must be moved just above or just below the *x*-axis; in this case we choose z=0.01 to enclose it in the studied portion of the plate. Second, the jump due to delamination is constrained to be j(x)>0, so when crack appears the symmetry of the vertical component of the displacement field between the auxiliary and real nodes is automatically lost due to the inelastic part.

Our studies show that, to deal with this problem, the most suitable way to enforce the physical boundary conditions is first to impose a null vertical displacement on the $\Gamma _1$ nodes, i.e.

19 $$\begin{aligned} u_{z.i}=0 \quad \quad \forall i=1,...,n_{\Gamma _1} \, . \end{aligned}$$

Then, the conditions on the auxiliary nodes should be set, but a straightforward definition is not possible due to a simple consideration on the degrees of freedom of the plate. Indeed, these latter are defined as the product of the coefficients of the ansatz polynomials and the number number of nodes along the *x*-axis, namely, $H/\Delta _x+1$, thus they are a well-defined amount. The constraint degrees come from the application of the Dirichlet boundary conditions, both the loading program on $\Gamma _4$, and the requirements on $\Gamma _1$ and $\Gamma _2$. If all of them are explicitly applied as restrictions for the ansatz, the structure results to be over-constrained and the results obviously turn out to be heavily affected by this. As the conditions on $\Gamma _1$ have shown to be the most impactful due to the different features of the boundary itself and to the loss of symmetry, these have been removed from the explicit formulation, and have been implicitly applied in the energy computation. This ensures the compliance with the requirement and avoids excessive restrictions on the displacement field. With respect to the kind of condition to be applied, there are two possible strategies. The first one is to impose that $u_x$ (the elastic part of $u_z$) of the auxiliary nodes is equal (equal and opposite) to that of the corresponding symmetric plate’s nodes. The second possibility is to chose a value for $u_x$ and $u_z$. To reduce the computational efforts, this latter choice was preferred, assigning $u_x=0$ and $u_z=0$, thus preventing any rigid body motion. With regards to the other boundaries, $\Gamma _3$ is a traction-free edge, while $\Gamma _4$ is subject to the application of the imposed displacement $u_z=\overline{u}$ through $t_f$ incremental steps of magnitude Δ.

## Results

The results obtained from the analysis of the plate described in Sect. 2, studied for different degrees of nonlocality, are reported in the following. Crack nucleation and its development, as well as the stresses [15, 24] in the plate, and the comparison with a local continuum. By assuming a uniform grid spacing $\Delta _x=\Delta _z=R/8=H/20$, the considered horizons are δ=3R/8 and δ=3R/4, where the *m*-ratios are 3 and 6, respectively. As exhaustively reported in the literature, the choice of m=3 is the most suitable to represent the local case in bond-based PD [39], while m=6 represents a more nonlocal case. The plates are characterized by having the same overall elastic stiffness. The analyses are performed in the small displacement regime, and the loading condition assumed is the imposed displacement mode. The value of the variable *h*(*x*) has been set a priori just above z=0 to foster nucleation approximately at the symmetry plane. At the same time, this avoids numerical problems given by the fact that the Heaviside function describing the delamination surface is not defined on the *x*-axis, and reduces the overall computational cost. On the one side, this choice represents a limitation to the possibility of developing the delamination surface at different values of *z*. On the other side, the onset of damage and fracture propagation have a well-established behavior in the problem of a plate with a central circular hole [32, 42]. Starting from this, the onset of damage is usually at the tip of the hole, or, more generally, where the stress concentration peak is located, and fracture propagates perpendicularly to the loading direction. In this fashion, the predetermined values of *h*(*x*) comply with these requirements.

**Fig. 6:**
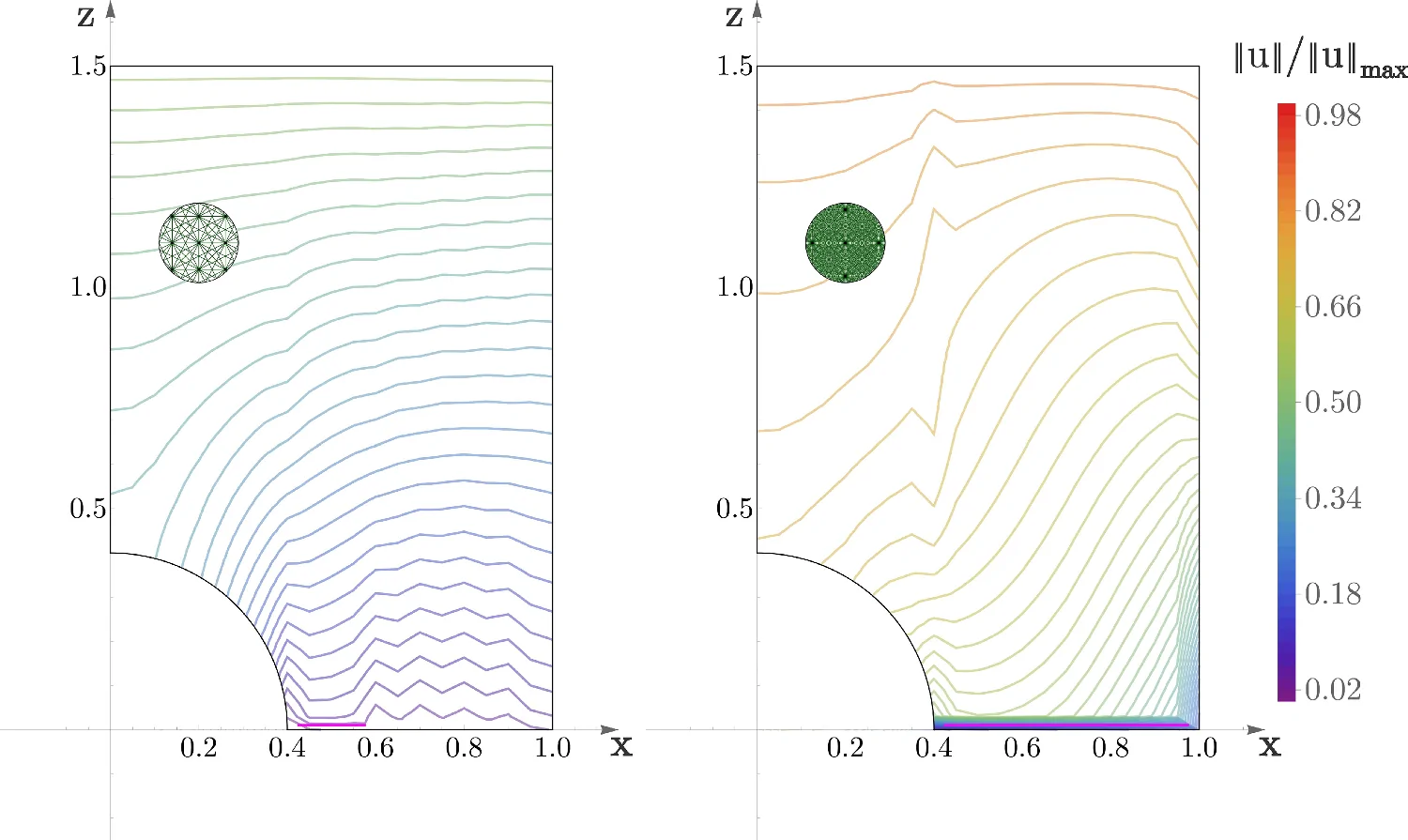
Representation of the normalized displacements’ magnitude and delamination surfaces for the two studied horizons. The considered loading steps correspond to the ones of the onset of damage. The delamination surfaces are represented by the continuous magenta lines

**Fig. 7:**
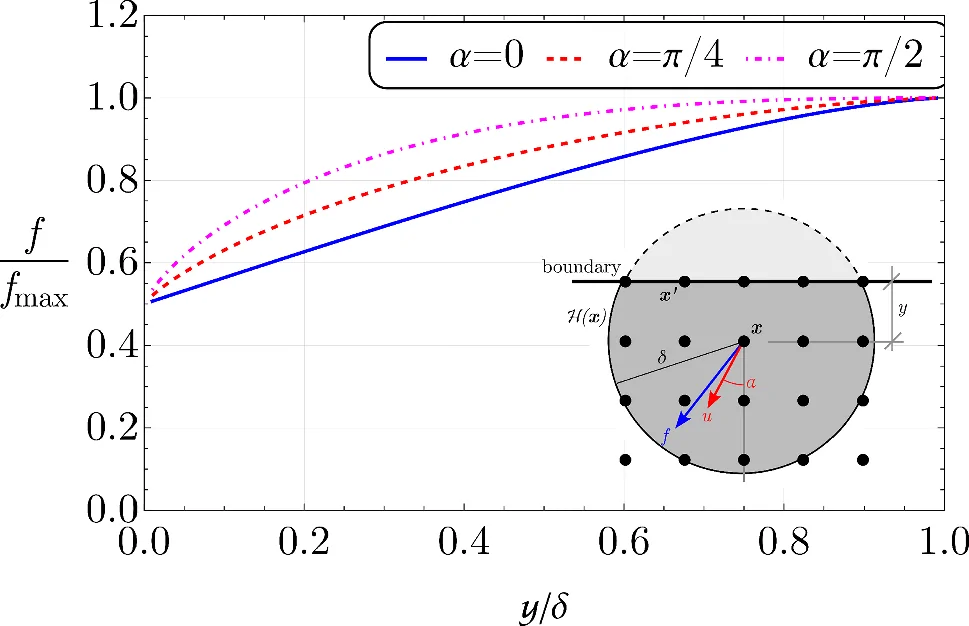
Measure of the force necessary to displace a peridynamic node, $\textbf{x}$, by a unit vector $\textbf{u}$, as a function of its distance to the physical boundary of the body. This quantity coincides with the integral of the peridynamic pairwise force function in the family of the point $\textbf{x}$ when the relative displacement between any couple of points satisfies $\boldsymbol{\eta } = \textbf{u}$

**Fig. 8:**
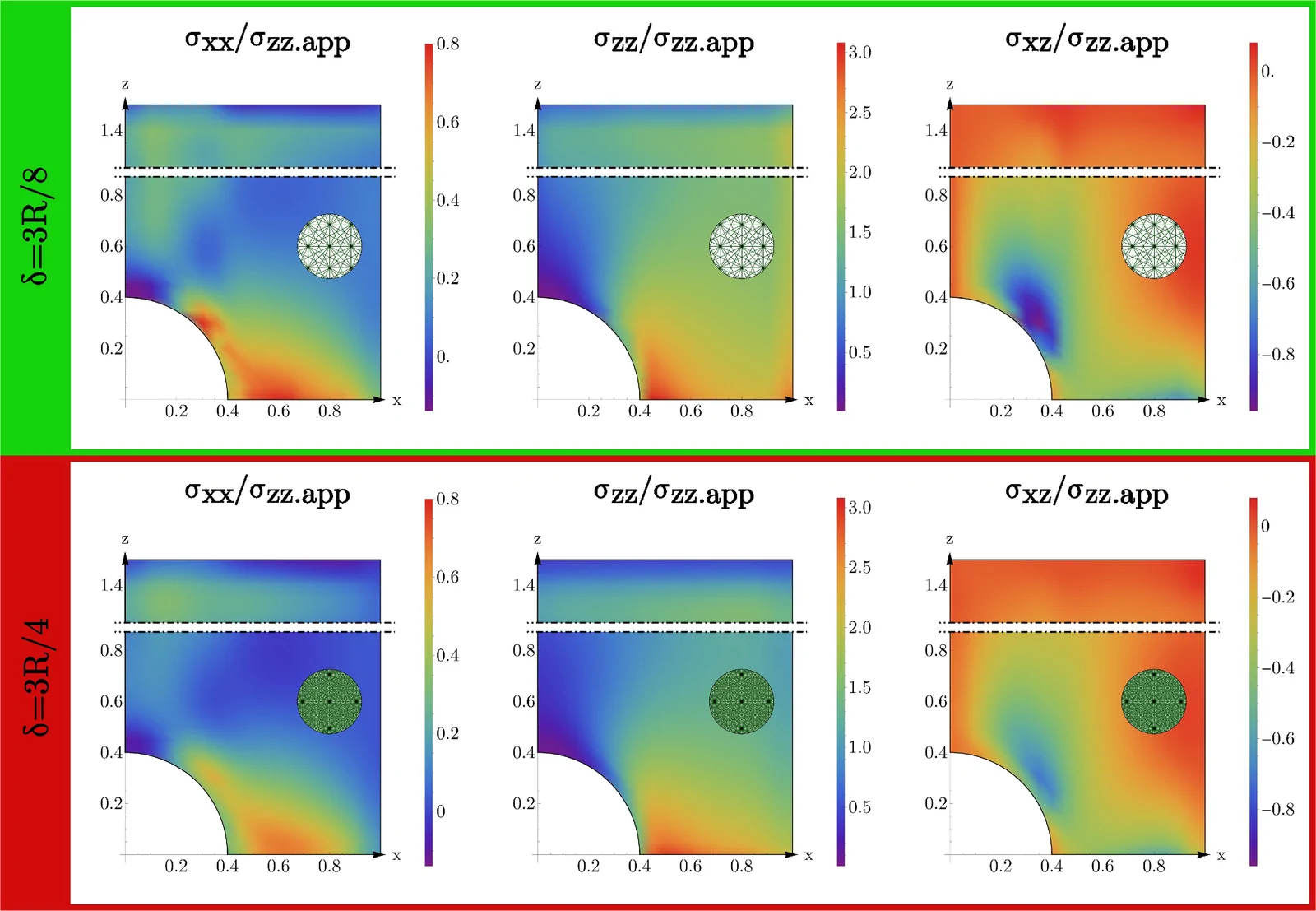
The graphs represent the distribution of the stresses normalized with $\sigma _{zz.app}$, i.e. the stress computed in a proper point in agreement with the stiffness considerations explained in this paragraph. All the results refer to the elastic regime. The first column shows the values of $\sigma _{xx}$. The second column represents the distribution of $\sigma _{zz}$, where the stress concentration factor is detectable, as well as the increasing distality and diffusion of the stress peak. The third column is a representation of $\sigma _{xz}=\sigma _{zx}$

**Delamination surface.** The main focus of this work is the study of crack nucleation and its development. For both the degrees of nonlocality studied, the onsets of damage are reported in Fig. 6. Through the use of a contour plot, the graphs show the magnitude of the displacement field normalized with respect to its maximum, while the delamination surface is represented by the continuous magenta lines. The loading steps represented in Fig. 6 are the ones associated to the onset of damage, so to the first appearance of crack. The most impactful result of this study is the different extension of the delamination surface at its onset (Fig. 7). Indeed by comparing the two pictures, one can notice that for the most nonlocal plate the magenta line, corresponding to a “jump” in the matter, extends from the tip of the hole to the boundary $\Gamma _3$. On the other side, the almost local setting shows a more localized damage, which is focused near the hole. These outcomes confirm the expectations, and are consistent with the results found in [8]. When the nonlocality increases, the number of interactions among the particles increases and distal nodes are involved, this impacts both the location of the fracture and the distribution of stresses, as highlighted in Fig. 8.

**Stresses and distality.** The procedure described in the previous paragraph has been used to retrieve the results reported in Fig. 8. It is worth noting that these outcomes and, in general, the whole work do not aim at approximate the local continuum results, but represent a study on the influence of the nonlocality of the continuum microstructure on a common and useful example of fracture mechanics. In this sense, the different choices of the horizon do not have to be regarded as a parametric study for defining the best internal length scale representing a local continuum. This is, instead, the analysis of two different materials with the same overall elastic stiffness, characterized by different degrees of nonlocality, which are responsible of the behavior of the plate during the fracture process.

In Fig. 8, the two rows represent the different horizons studied, i.e. the green row corresponds to the most local case, while the red one stands for the more nonlocal one. The normal stress on the *x*-axis can be read in the first column, the normal stress on the *z*-axis in the second column, while $\sigma _{xz}$ is represented in the third column. The results are normalized with the applied stress along *z*, i.e. $\sigma _{zz.app}$, both to perform a consistent comparison between the outcomes of different nonlocalities, and to retrieve the stress concentration factors. For each horizon considered, the choice of the $\sigma _{zz.app}$ value is not trivial due to the great variation of the local stiffness in the area where the imposed displacement is applied. Indeed, due to the fact that the plate has not infinite length in the loading direction, there is a lack of homogeneous stressed zone where the normalization value is usually assumed as the resultant reaction force divided by the loading area. In absence of this, the Authors choose to normalize the stresses using two different values of $\sigma _{zz.app}$, both referred to nodes with the same stiffness. First, a consideration on the stiffness must be done. In this case, the concept of stiffness refers to a scalar mechanical indicator introduced to quantify the geometrical weakening experienced by a PD node when its family is truncated by the physical boundary of the body. Specifically, let $\textbf{x}$ be a material point and let $\mathcal {H}(\textbf{x})$ denote its family, i.e., the set of points lying within the interaction horizon δ from $\textbf{x}$. When $\textbf{x}$ is sufficiently far from the boundary, $\mathcal {H}(\textbf{x})$ is the full circular neighborhood. On the contrary, when $\textbf{x}$ approaches the boundary, $\mathcal {H}(\textbf{x})$ is no longer complete and progressively changes shape from a full circle to a circular sector. This geometrical reduction of the family directly implies a reduction in the number of available interactions, and therefore in the force required to impose a given displacement to that point while all the other points are kept fixed.

For this reason, here the stiffness is introduced as the magnitude of the force needed to displace point $\textbf{x}$ by a unit amount while the surrounding points are fixed. In symbolic form, such force can be written as

20 $$\begin{aligned} \textbf{f}(\textbf{x})=\int _{\mathcal {H}(\textbf{x})} \mathbb {C}\,\boldsymbol{\eta }\, dV' =\left[ \int _{\mathcal {H}(\textbf{x})} \mathbb {C}\, dV' \right] \textbf{u}(\textbf{x}) =\textbf{A}(\mathcal {H})\,\textbf{u} \; , \end{aligned}$$

where $\textbf{u}$ is the imposed displacement of the node, $\boldsymbol{\eta }$ is the corresponding relative displacement field, and $\textbf{A}(\mathcal {H})$ is a second-order tensor depending on the shape of the family $\mathcal {H}(\textbf{x})$, that is, on the integration domain itself. The analogy with the force-displacement relation of a discrete lattice system is the reason why the term stiffness is adopted.

When the family is complete, namely when the point lies at a distance from the boundary larger than the horizon, $\textbf{A}(\mathcal {H})$ is isotropic, i.e., proportional to the identity tensor. In that case, the resulting force is always aligned with the imposed displacement, and the stiffness can be unambiguously measured through the ratio between the moduli of force and displacement.

When the family is truncated, however, $\textbf{A}(\mathcal {H})$ is no longer isotropic. In particular, for a circular-sector-shaped family, $\textbf{A}(\mathcal {H})$ possesses principal directions associated with the boundary cut and with the orthogonal direction. As a consequence, for a generic imposed displacement, the force vector is in general not parallel to the displacement vector. Therefore, in this boundary region, the quantity used here should be regarded as a scalar proxy of a genuinely tensorial directional stiffness, rather than as a complete descriptor of the local mechanical state. In particular, the adopted scalar measure is the ratio between the magnitudes of $\textbf{f}$ and $\textbf{u}$, normalized with respect to the corresponding value in the bulk, i.e., for a node with complete family.

The linear variation of the stiffness in the upper strip of the plate is a physically reasonable approximation of the boundary weakening effect, and based on the analysis shown in Fig. 7.

In particular, when the imposed displacement is purely vertical (case α=0, blue solid curve in the Fig. 7), the normalized scalar stiffness defined above exhibits an almost linear variation over most of the interval $0 \le y/\delta \le 1$, except in the portion very close to y/δ=1, where the transition to the bulk behavior becomes smoother. Since the purpose of this construction is to provide a practical and sufficiently accurate criterion to identify mechanically corresponding nodes in the two δ-cases analyzed, the linear interpolation appears as a reasonable and effective approximation. Thus, by assuming that the rows of nodes enclosed in $z \ge (L-\delta )$ and z≤L satisfy a linear law of local stiffness variation, we can find the relation among the equivalent stiffness of the case with horizon δ=3R/8 and δ=3R/4. Following this, it is possible to determine the rows of nodes where the local stiffness of the two cases are comparable and, among the rows’ nodes, we have chosen to normalize the stiffness with the stress associated to the nodes in x=0, so on the symmetry axis of the plate. This latter choice ensures to avoid any influence of the $\Gamma _3$ boundary effect due to the incomplete horizons and consequent stiffness reduction, while it is worth noting that no boundary effects are associated to $\Gamma _2$, because this is a fictitious edge, and its stiffness has been restored with auxiliary nodes. So for the case of δ=3R/8, $\sigma _{zz.app}$ is computed in the node $\{0,1.25\}$, while for δ=3R/4 the chosen node is $\{0,1.35\}$. All the distributions of stresses considered confirm the monotonic increase of fracture distality with the increasing nonlocality. Indeed, the representations of $\sigma _{zz}$ on the first row and second column shows that the peak of stress, and so the fracture, in the most local case is near the hole and approximately concentrated in the area from x=0.425 and x=0.575. On the opposite side, in the second row the distribution of $\sigma _{zz}$ for the nonlocal case is slightly different. The peak is more diffuse, starting from x=0.425 and reaching x=0.90, and this is also confirmed by the fact that the stress concentration factor is smaller than the one of the almost local case. This suggests that a higher degree of nonlocality in plates with equal initial stiffness leads to a better redistribution of the concentration of stress, causing non-trivial onset of fractures. These results also corroborate the displacements outcomes presented in Fig. 6, where the delamination surface becomes more diffuse as the nonlocality increases.

**Fig. 9:**
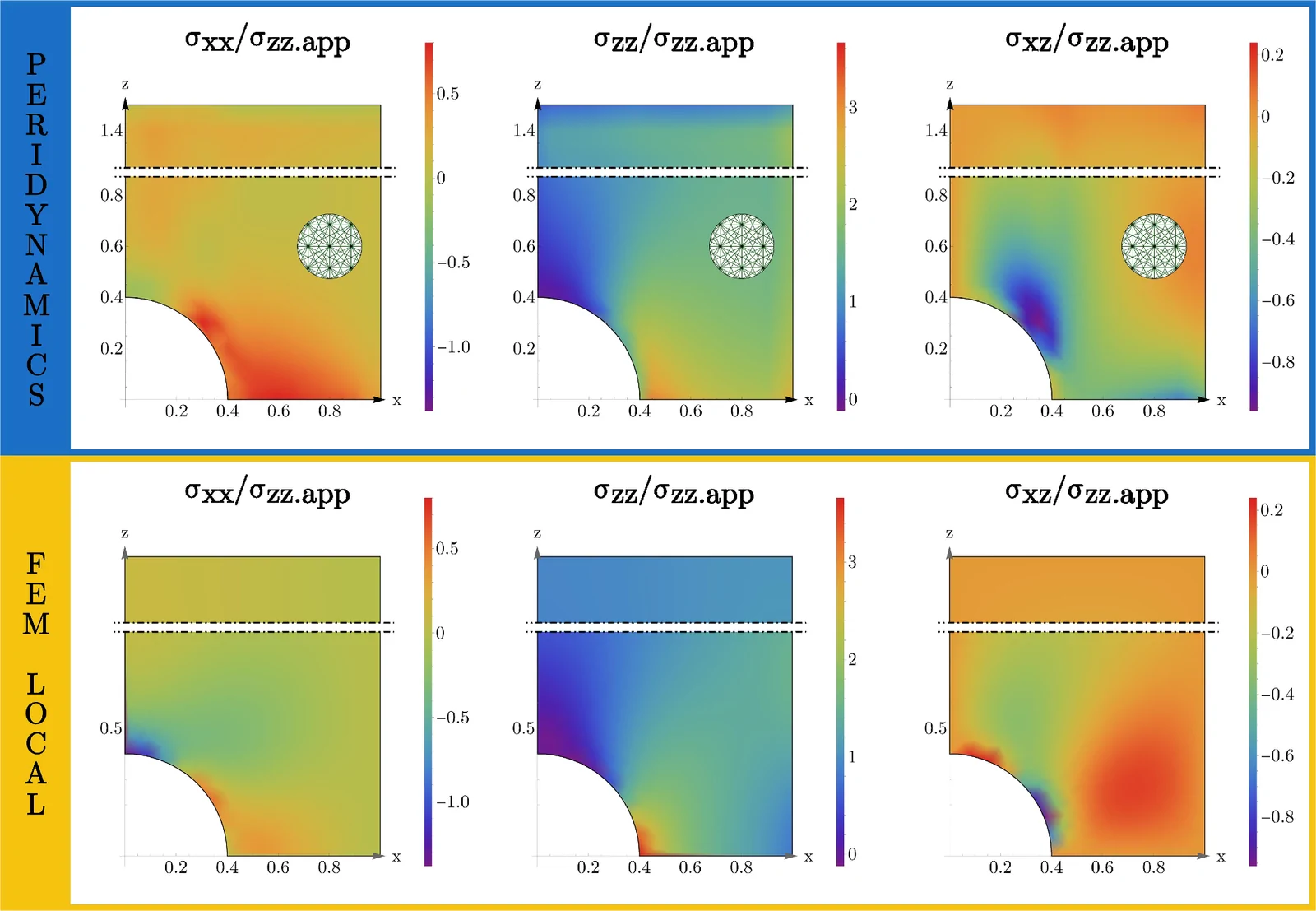
The graphs represent the comparison of the normalized stresses for the most local PD case and the local continuum. The grid spacing for the PD model is $\Delta _x=\Delta _z=R/8$, and the horizon is assumed to be δ=3R/8 so the *m*-ratio is 3

**Comparison with local continuum.** The stress computation approach is validated by verifying that the stresses of the most local case correspond to the ones retrieved with a FEM analysis for the local plate described by CCM, see Fig. 9. The FEM study has been carried out by modeling the local plate with central circular hole . The mechanical parameters have been directly related to the PD ones, and the analysis has been performed in the sole elastic regime, as the purpose was to obtain benchmark results for the stress distribution and concentration factor. It is worth noting that the possibility of recovering the CCM local results with the most local PD plate under consideration depends both on the choice of the internal length scale and on the grid spacing (i.e $\Delta _x$ and $\Delta _z$) [39, 43]. As exhaustively reported in [7, 17], the convergence to classical continua is ensured not only through the δ-*convergence*, needed to approximate the horizon to the local one, but also via the *m*-*convergence*. Indeed, the sole *m*-*convergence* (where δ is fixed and $m \rightarrow \infty$) only ensures that the numerical peridynamic approximation converges to the exact one for a given value of the horizon. On the other side, the sole δ-*convergence* (where $\delta \rightarrow 0$ and *m* is fixed or increases, at a slower rate, with decreasing δ) leads to an approximation of the local solution that can potentially be non uniform. The correct procedure which ensures the uniform convergence of the numerical peridynamic solution to the exact local one is the (δm)-*convergence*, where δ decreases while *m* increases faster than this. It should also be noticed that, in general, the value of the horizon cannot be equal to the mesh size for mechanical reasons [7, 43]. Thus, in the case of bond-based PD it is common to adopt the ratio m=3 to best fit the local results [39]. This is the chosen value also for this work, where the PD plate used for the comparison with CCM results is the one with horizon $\delta =3\Delta _x=3R/8$. It can also be noted that here the assumption of m=3 is not correlated with specific convergence procedure, as the discretization grid is kept unchanged. This choice is in agreement with the whole study, where the purpose is to analyze the role of different microstructures in the behavior of the plate, and not to mimic a local continuum. Nevertheless, the comparison of the outcomes of the most local structure analyzed and the benchmark ones of CCM is meaningful. Actually, the results reported in Fig. 9 validate the model for this case of study, as the stress concentration factor almost reaches the value retrieved in a local FEM simulation. This is also confirmed by the analytical computation of the stress concentration factor of a finite width plate with a central circular hole subject to an imposed tensile traction, see Appendix A. However, it is worth noting that the PD results show little differences with the local ones. The reason of this can be ascribed both to the choice of using an averaged measure of the PD stress, and to the fact that in this work we did not use any of the strategies needed for restore the local conditions near the boundaries.

A summary of the stress concentration factors obtained for both the PD cases analyzed and for the FEM local continuum are reported in Table 1.

## Conclusions

In this work, the influence of the degree of nonlocality on the onset of damage and stress distribution in a classical fracture mechanics problem is analyzed.

These investigations have been pursued by making use of the PD theory [38, 39], which naturally allows to deal with nonlocal interactions. The model used is capable of determining crack nucleation and propagation thanks to the choice of the displacement field, therefore it is suitable for studying fracture in a plate with a central circular hole. The set up of the problem is that of a finite width plate with such a hole subject to tensile traction, applied through suitable imposed elongations, for which there are well-known analytical and numerical results in the literature. *The aim is to investigate if any differences arise in the mechanical response of the studied plate when the internal length scale of the medium changes*. To this extent, the model has been tested for two different horizons, which is the PD definition of the degree of nonlocality, and preserving the same overall elastic stiffness. It is worth mentioning that, although the initial stiffness of the plates analyzed is tuned to be the same, their mass cannot be equal due to the fact that a non-homogeneous transformation is applied.

**Table 1:** Estimates of the stress concentration factors for the tractioned nonlocal peridynamic plate with central hole. Here, “Local” refers to the value of the stress concentration factor for a local elastic plate

Case	Horizon	$\textbf{m}$-ratio	$\sigma _{zz}/\sigma _{zz.app}$
FEM	Local continuum		3.66
PD	δ=3R/8	m=3	3.08
PD	δ=3R/4	m=6	2.95

The obtained results show that an increase in the distality of fracture nucleation is predicted by the model as the degree of nonlocality increases. This confirms that longer interactions among the plates’ nodes correspond to unconventional onset of damage, which moves away from the tip of the hole. The internal length scale of the medium plays a crucial role also in the stress distribution and, primarily, in the value of the stress concentration factor. In order to delve in deeper into this topic, first an analysis on the actual meaning of PD stress has been conducted. In the specific context of a discretized PD medium, the continuum PD stress tensor proposed in [24] must be adapted. A “geometrical approach” has been chosen in this case, the plate has been discretized through a tessellation, and each derived area is represented by its central point. The stresses in these points are computed by considering and averaging the contribution of all the bonds crossing their corresponding area. In this fashion, a discretized version of the PD stress has been derived, and we demonstrated that this kind of approach is equivalent to that used by Fallah and coauthors in [15]. It is worth noting that such a method does not consider if the node is in a central position of the plate or nearby its edges, thus there are no differences between nodes with complete or incomplete horizons.

By analyzing the results retrieved with this method, the following conclusions can be drawn: (i) the major stress concentration is located in proximity of the onset of damage, whose site is unconventional due to the choice of the nonlocality degree; (ii) the value of the stress concentration factor decreases with increasing nonlocality (see Table 1) and the stress peak becomes more diffuse along the *x*-axis; (iii) the local behavior is retrieved when the smaller horizon is used, although the use of an averaged measure. For what concerns the point (i), the more stressed zone moves slightly away from the tip of the hole following the pattern of increasing fracture distality with increasing horizon, as expected due to its general relation with the onset of fracture, and its specific relation due to the stress-based failure criterion chosen for the bonds. As stated in (ii) then, the value assumed by the stress concentration factor decreases with increasing nonlocality, and this is related to the higher diffusion of the peak of stress. For this reason, the measure gives useful mechanical information on the plate and, as pointed out in (iii), it has been verified that it provides results convergent to the local case when the smaller horizon is taken into account.

The results retrieved in this work support the possibility of using nonlocality as a design strategy for obtaining plates with specific behaviors. Indeed, by tuning the internal length scale of the medium, it is possible to both move the onset of fracture away from its classical position and change the value of the stress concentration factor ultimately affecting also toughness, an aspect of great interest in material design and engineering [14]. On the other hand, as underlined above, these outcomes are subject to changes of the total mass of the plate. In the next future, an important step for a greater in-depth analysis on nonlocal plates behavior will be the study of plates with several degrees of nonlocality, different *R*/*H* and R/δ ratios, and constant overall mass. Furthermore, a consistent comparison with the results of the quantized fracture mechanics (QFM) [34, 35] might be relevant for finding possible analogies with other (possibly discrete) versions of classical continua.

## Data Availability

The data generated during the current study are available from the corresponding author on reasonable request.

## Stress Concentration Factor of the Local Continuum

Starting from the Kirsch equations, written in polar coordinates [22], for the stress field in an infinite plate with a circular hole of radius *R* and applied stress $\sigma _{\infty }$, namely:

21 $$\begin{aligned}&\sigma _{rr}=\frac{\sigma _{\infty }}{2} \left( 1-\frac{R^2}{r^2}\right) +\frac{\sigma _{\infty }}{2}\left( 1-4\frac{R^2}{r^2}+3\frac{R^4}{r^4}\right) \cos {2\theta } \; , \end{aligned}$$

22 $$\begin{aligned}&\sigma _{\theta \theta }=\frac{\sigma _{\infty }}{2}\left( 1+\frac{R^2}{r^2}\right) -\frac{\sigma _{\infty }}{2}\left( 1+3\frac{R^4}{r^4}\right) \cos {2\theta } \; , \end{aligned}$$

23 $$\begin{aligned}&\sigma _{r\theta }=-\frac{\sigma _{\infty }}{2}\left( 1+2\frac{R^2}{r^2}-3\frac{R^4}{r^4}\right) \sin {2\theta } \; , \end{aligned}$$

one can notice that $\sigma _{\theta \theta }$ in $\theta =\pi /2$ corresponds to the stress $\sigma _{zz}$ considered in this work, and that $\sigma _{\infty }=\sigma _{zz.app}$. In this fashion, the value of the tensile stress associated to the node $\{x,z\}=\{R,0\}$ in the case of an infinite plate is $\sigma _{zz}=3 \; \sigma _{zz.app}$, where the stress concentration factor is $K_t=3$. In the current case of study, the finite width of the plate must be taken into account. This can be done by considering a correction factor for $K_t$, as reported in [32]:

$$\begin{aligned}&K_t=K_{g} \left( 1-\frac{R}{H}\right) \; ,  &   \text {where} \; \; \; K_g=\frac{\sigma _{max}}{\sigma _{zz.app}} \; . \end{aligned}$$

Due to the above described modulation, in this case the ratio between the maximum tensile stress in the plate and the applied stress is given by $K_g$, which can be expressed through the following relation, [32]:

24 $$\begin{aligned} K_g=0.284+\frac{2}{1-\frac{R}{H}}-0.600\left( 1-\frac{R}{H}\right) +1.32 \left( 1-\frac{R}{H}\right) ^2 \; . \end{aligned}$$

For the actual ratio among the diameter of the hole and the width of the plate under exam, e.g. R/H=0.4, this coefficient evaluates to $K_g=3.73$, and the final relation among the two stresses is $\sigma _{zz}=3.73 \; \sigma _{zz.app}$.
